# Emphysematous prostatic abscess due to candidiasis: A case report and review of the literature

**DOI:** 10.1097/MD.0000000000019391

**Published:** 2020-02-28

**Authors:** Zhongyi Li, Jiaming Wen, Nan Zhang

**Affiliations:** Department of Urology, the Second Affiliated Hospital of Zhejiang University School of Medicine, Hangzhou, Zhejiang Province, China.

**Keywords:** candidiasis, diabetes mellitus, emphysematous prostatic abscess

## Abstract

**Rationale:**

The emphysematous prostatic abscess is a rare but potentially life-threatening clinical condition. The early diagnosis is difficult due to nonspecific symptoms.

**Patient concerns:**

A 72-year-old man with poorly controlled diabetes mellitus was admitted to hospital because of dysuria and acute urine retention. He had a refractory fever after admission.

**Diagnoses:**

The diagnosis of emphysematous prostatic abscess was confirmed by culture of catheterized urine and pelvic CT.

**Interventions:**

We tried to give antimicrobial treatment and control of blood glucose at first, but the infection could not be controlled by antibiotic therapy and control of blood glucose. TRUS-guided aspiration was performed, but obviously not adequate for abscess drainage and the abscess progressed. TUR was then performed and the infection was gradually controlled.

**Outcomes:**

Pelvic CT scan 1 month after discharge showed complete resolution of the EPA.

**Lessons:**

Given the poor conservative treatment effect of emphysematous prostatic abscesses, CT or TRUS should be performed in the patients with suspected diagnosis. Early and appropriate drainage with proper antibiotic therapy is important to achieve a favorable outcome.

## 1 Introduction

Emphysematous prostatic abscess (EPA) is a rare disease, characterized by localized collection of gas and purulent exudates in the prostate gland. The first case was reported in 1983^[1]^ and only a few cases have been reported since then. The pathogens causing EPS included various bacterial and fungal organisms, and the most commonly reported microorganism causing EPA was *Klebsiella pneumoniae*.^[2]^ The management of emphysematous prostatic abscess is not standardized due to the limited number of cases reported. Herein, we present a rare case of emphysematous prostatic abscess due to *Candida tropicalis* in a patient with poorly controlled diabetes mellitus and a review of the literature.

## 2 Case presentation

The patient provided informed consent for the publication. The study was approved by the ethics institutional review board of the Second Affiliated Hospital of Zhejiang University School of Medicine.

**Figure 1:**
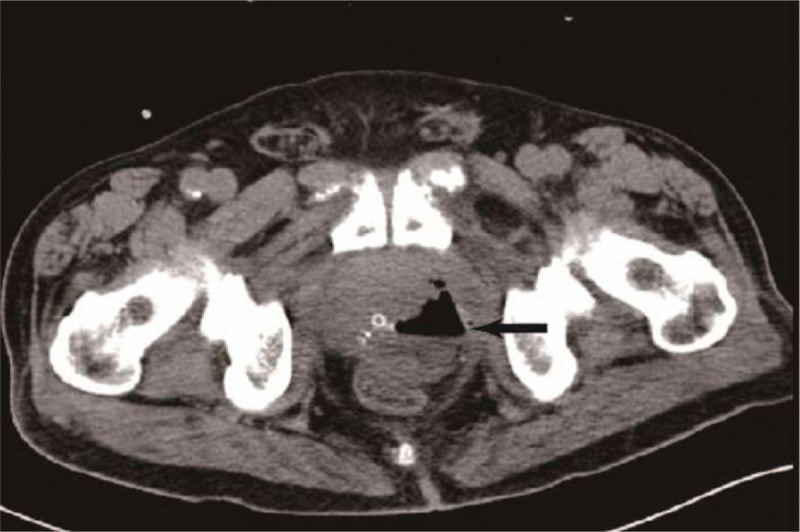
Pelvic CT revealed a collection of gas and purulent exudates in the prostate gland (arrow) on day 8 of admission (before aspiration).

**Figure 2:**
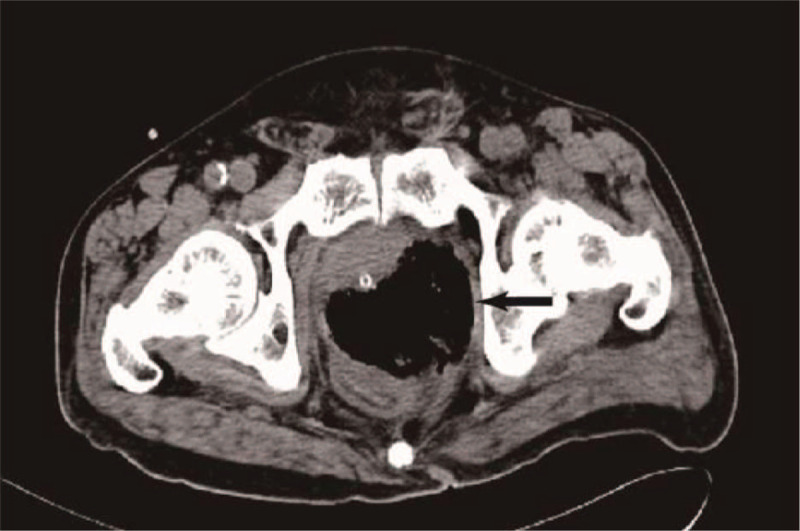
Pelvic CT revealed a progress of gas and purulent exudates collection in the prostate gland (arrow) on day 14 of admission (after aspiration).

**Figure 3:**
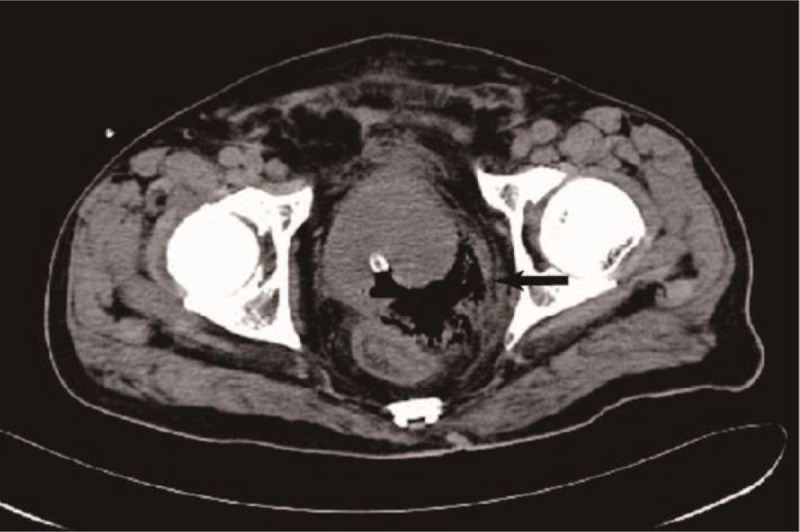
Pelvic CT revealed an improvement of EPA in the prostate gland (arrow) on day 20 of admission (after TUR).

**Figure 4:**
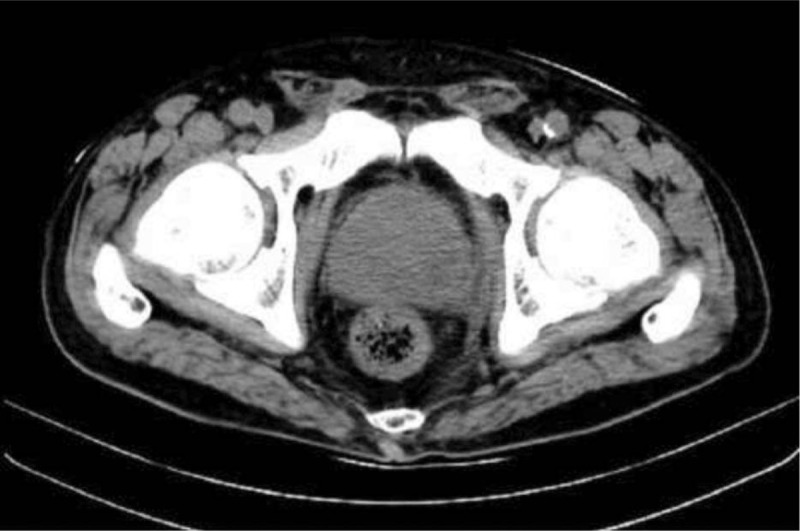
Pelvic CT showed almost complete resolution of the EPA 1 month after discharge.

A 72-year-old man was admitted to our department because of dysuria for 5 months, and acute urine retention for 6 days prior to the admission. The ultrasonagraphy of the prostate showed an enlarged prostate (5.71 × 5.52 × 5.38 cm, without sign of abscess), and transurethral Foley catheter was inserted and kept in place in the emergence department. The patient had a history of type 2 diabetes mellitus for over 10 years. Digital rectal examination revealed a mild, enlarged prostate, with no local tenderness. On the day he was admitted, the body temperature was 37.8°C. Laboratory tests showed a white blood cell count of 8.6 × 10^9^/L with 73.1% neutrophils, hemoglobin 144 g/L, alanine aminotransferase (ALT) 11 IU/L, aspartate aminotransferase (AST) 12 IU/L, blood urea nitrogen (BUN) 4.59 mmol/L, fasting glucose 15.09 mmol/L, prostate specific antigen (PSA) 16.023 ng/ml. Urinalysis showed white blood cells 31 /μL, red blood cells 449/μL, presence of glucose (4+). We adjusted the oral hypoglycemic agents (OHA) and monitored blood glucose. On day 3, the patients had chills and the body temperature was 39°C. Laboratory tests showed a white blood cell count of 14.1 × 10^9^/L with 82.7% neutrophils, C-reactive protein (CRP) 190.2 mg/L. Blood sample was taken for culture immediately. Empiric antimicrobial treatment with intravenous cefoperazone/sulbactam (1:1) 2.0 g was administered every 8 hours. On day 4, *Candida tropicalis* was isolated from the culture of catheterized urine. Fluconazole injection 200 mg every 12 hours was added. But state of high fever seemed no improvement. On day 8, CT of the pelvis was performed, and revealed swelling of the prostate with air and fluid accumulation (4.5 × 3.5 cm), suggestive of EPA (Fig. 1). On day 9, transrectal ultrasound guided prostate abscess aspiration was performed. Only 5 ml reddish purulent fluid was extracted, saline solution wash did not help to extract more purulent fluid. The blood culture taken before and pus culture both were negative. The body temperature seemed improving after the aspiration, but he still got low-grade fever. On day 14, CT of the pelvis was performed, and gas formation was even bigger (75mm × 59 mm) in the prostate gland (Fig. 2). Laboratory tests showed a white blood cell count of 25.9 × 10^9^/L with 88.3% neutrophils, CRP > 270 mg/L. So transurethral unroofing of prostatic abscess was performed immediately. A suprapubic cystostomy was performed during the surgery for urinary diversion. There was not so much purulent fluid in the cavity of abscess, but lots of necrotic tissue around the abscess cavity. Cefoperazone/sulbactam and fluconazole were administered continuously after the surgery. On day 20, he had no fever, and pelvic CT scan was rechecked showing a great improvement in the size of the abscess cavity within the prostate gland (Fig. 3). He was discharged on day 22. Parenteral antibiotics of fluconazole were kept for 14 days after discharge. Pelvic CT scan 1 month after discharge showed complete resolution of the EPA (Fig. 4). The cystostomy tube was removed 4 weeks later.

## 3 Discussion

Prostatic abscess is a rare complication of acute bacterial prostatitis, reported in 0.5% to 2.5% of patients presenting with inflammatory prostatitis.^[3]^ Emphysematous prostatic abscess is a particularly rare form of prostatic abscess, which is characterized by localized collection of gas and purulent exudates in the prostate gland. The classical symptoms and signs of emphysematous prostatic abscess include dysuria, fever, odynuria, increased frequency and urgency to urinate, urinary retention, perineal pain, and fluctuance on digital rectal examination. The early diagnosis is difficult due to nonspecific symptoms. Patients are often initially treated for prostatitis, with a median delay in a correct diagnosis of 8 days.^[2]^ Imaging techniques such as pelvic computed tomography (CT) and transrectal ultrasonography (TRUS) are most valuable for detecting EPA.

We searched PubMed for English language publications using the keywords “emphysematous prostatitis” or “emphysematous prostatic abscess” for the period 1983 to 2019. Sixteen cases with EPA were included in 14 reports. We reviewed the demographic characteristics, age, underlying disease, pathogen, management, and outcomes of the 16 patients with EPA (Table 1). Most of the patients were from Asia (87.5%, 14/16). The median age of patients was 62 years old (range, 45–81 years). 14 (87.5%) had diabetes mellitus. Various bacterial and fungal organisms were isolated in urinary and/or pus cultures, with *K pneumoniae* being the most prevalent pathogen (50%, 8/16). Other organisms reported included *Escherichia coli* (25%, 4/16), *Candida species* (18.75%, 3/16), *Pseudomonas aeruginosa*, *Bacteroides fragilis*, and *Citrobacter species*. Two cases (12.5%) had mixed infections. Fifteen (93.8%) underwent drainage of various ways. Except for one (with DM, dementia, advanced gastric cancer) had 8-week antimicrobial therapy, and he was transferred to another hospital for rehabilitation when condition improved, with no further information. Seven of 16 patients (43.8%) underwent a suprapubic cystostomy. The mortality rate was 18.75% (3/16).

**Table 1:**
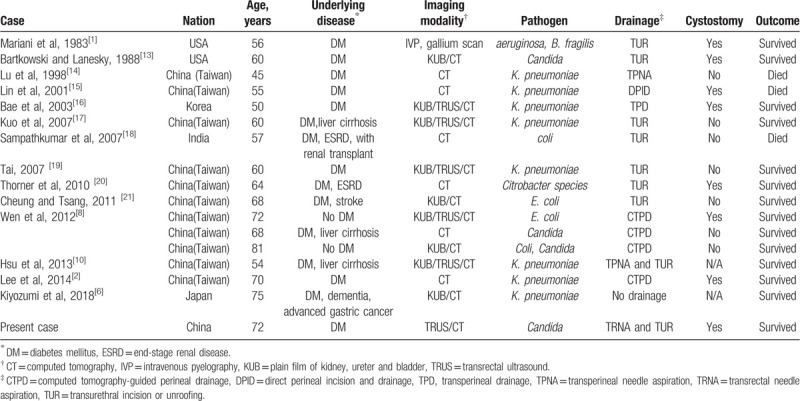
Cases of emphysematous prostate abscess, including 16 reported patients and the present case.

EPA appears to be geographically more common in Asia, and different from other forms of prostatitis, *K pneumoniae* rather than *E coli* appears to be the most common causative pathogen.^[4–6]^ Whether some virulent strain of *K pneumoniae* or host factor resulted in the phenomenon deserves further investigation.^[7,8]^ In this case, we determined EPA was due to *C tropicalis* infection. The diagnosis was confirmed by pelvic CT, with about 8 days delay. *C species* also seems to be more prevalent in EPA (23.5%, 4/17, include this case) than in other urinary tract infections. However, due to the small number of cases reported, further study is warranted.

Prostatic abscess may develop secondary to reflux of infected urine into the prostate or from hematogenous dissemination.^[5,9]^ The risk factors for prostatic abscess formation include bladder outlet obstruction, urethral manipulation and systemic disease such as diabetes mellitus, liver cirrhosis and other immune-compromising conditions.^[2,5,8]^ In this case, the patient had poorly controlled diabetes, together with urinary tract obstruction and catheterization may be important risk factors that contributed to prostatic abscess formation.

The treatment of choice is prompt and thorough abscess drainage with early antibiotic therapy and strict control of blood glucose. Abscess drainage may be performed by transurethral or transperineal way. Open surgery is less recommended nowadays. Transurethral incision or unroofing can provide complete drainage but it also increases the risk of sepsis.^[8,10]^ Hydraulic pressure during the transurethral procedure may push pathogens into the systemic blood stream and lead to sepsis or septic shock. The surgery should be done in selected patients who are hemodynamically stable and able to tolerate the anesthesia, and best to be quick and effective. The transperineal route is safer owing to its application under local anesthesia, but the disadvantage is possible incomplete drainage, abscess recurrence and longterm catheter indwelling needed. Because ultrasound waves are reflected by gas, CT-guided transperineal abscess drainage for emphysematous prostatic abscess may be more precisely and recommended.^[4,8]^ For other forms of prostate abscess, based on outcomes from several large case series studies, TRUS-guided aspiration rather than indwelling drainage is considered the standard treatment before progressing to other therapies.^[5,11,12]^ In the case we presented, TRUS-guided aspiration was obviously not adequate for abscess drainage, and the infection was not controlled until TUR was performed. We assume that the air, thick purulent exudates and necrotic tissue of EPA made it difficult to drain by aspiration and much easier to recur.

## 4 Conclusion

In conclusion, emphysematous prostatic abscess is a rare but highly morbid infectious disease that occurs in immuno-compromised patients, especially with diabetes mellitus. CT or TRUS should be performed in the patients with suspected diagnosis. Early and appropriate drainage with proper antibiotic therapy is important to achieve a favorable outcome.

## Author contributions

**Data curation:** Jiaming Wen.

**Project administration:** Jiaming Wen.

**Supervision:** Nan Zhang.

**Writing – original draft:** Zhongyi Li.

**Writing – review & editing:** Nan Zhang.

Zhongyi Li orcid: 0000-0001-8139-9137.
